# Psoriasis-Specific RNA Isoforms Identified by RNA-Seq Analysis of 173,446 Transcripts

**DOI:** 10.3389/fmed.2016.00046

**Published:** 2016-10-07

**Authors:** Sulev Kõks, Maris Keermann, Ene Reimann, Ele Prans, Kristi Abram, Helgi Silm, Gea Kõks, Kulli Kingo

**Affiliations:** ^1^Department of Pathophysiology, Centre of Translational Medicine, University of Tartu, Tartu, Estonia; ^2^Department of Reproductive Biology, Estonian University of Life Sciences, Tartu, Estonia; ^3^Department of Dermatology, University of Tartu, Tartu, Estonia; ^4^Department of Dermatology, Tartu University Hospital, Tartu, Estonia

**Keywords:** psoriasis, transcriptome, transcript expression profiling, RNA sequencing

## Abstract

**Background:**

Several studies have been published that investigated potential links between transcriptome changes and psoriasis using microarrays and RNA-seq technologies, but no previous study has analyzed expression profile of alternatively spliced transcripts in psoriasis.

**Objectives:**

Identification of potential alternatively spliced RNA isoforms with disease-specific expression profile.

**Methods:**

Using our published RNA sequencing data from lesional psoriatic (LP), non-lesional psoriatic (NLP), and normal control skin (C), we analyzed the differential expression of RNA splicing variants. LP sample was compared with NLP, as was LP with C and NLP with C.

**Results:**

Transcript-based annotation analyzed 173,446 transcripts (RNA isoforms), and around 9,000 transcripts were identified as differentially expressed between study groups. Several previously undescribed RNA variants were found. For instance, transcript ETV3_3 (ENST00000326786) was significantly downregulated in LP and NLP skin. ETV3 is a transcriptional repressor that contributes to the downstream anti-inflammatory effects of IL-10. We also identified diseases-specific transcripts (S100A7A, IL36RN_4, and IL36G_3) of genes already recognized to be involved in inflammation and immune response.

**Conclusion:**

Psoriasis is characterized by significant differences in the expression of RNA alternative isoforms. Description of these new isoforms improves our knowledge about this complex disease.

## Introduction

Psoriasis is one of the most prevalent chronic inflammatory diseases that affect the skin and joints (1). The disease affects 2–3% of the worldwide population, although the clinical course and severity varies (2). The cause of psoriasis is unknown; it is a complex disease with multifactorial pathogenesis where several genes interact and eventually induce the active disease (3–5).

Studies of the psoriatic gene expression profiles using microarrays and RNA-seq technologies have revealed large number of differentially expressed genes (DEGs) in lesional skin (6–12). More than 1,000 differentially regulated genes have been found for psoriatic compared to non-psoriatic skin using microarrays (7, 8). These studies have resulted in identification of new gene candidates regarding the pathogenesis of psoriasis. Around 80% of significantly elevated genes in psoriasis lesions are related to keratinocyte activity and infiltration by T-cells and macrophages (10). In addition to GeneChips, recent data from RNA-seq provide a more comprehensive overview of the transcriptional landscape of psoriasis. RNA-seq is able to detect transcripts and splicing forms undetectable with other tools (13). Transcriptome-based classification is able to discriminate subgroups of psoriasis with different molecular patterns (14). Several RNA-seq based studies on psoriasis have been published to date (11, 12, 15). All these studies described some previously undiscovered RNAs involved in psoriasis, supporting the power of RNA-seq to discover novel genes. In one study, three pairs of lesional and normal skin samples from psoriatic individuals were analyzed (15). Li et al. (11) found 3,577 DEGs between lesional and normal skin using biopsies from 92 psoriatic patients and 82 normal individuals. In a recent study, we performed RNA-seq-based gene expression profiling on 12 paired lesional (LP) and non-lesional (NLP) skin samples from psoriatic individuals and 12 skin samples from controls (C) (12). Besides other well-known genes, we identified robust expression of IL36 cytokines to be involved in the clinical development of psoriasis.

Most of these earlier studies focused only on gene-level annotation and as a result, transcript-specific information was never used. In one study however, exon-specific differential analysis was applied and 343 differentially used exons in lesional skin were discovered compared to controls (11). This finding suggests that transcript-based annotation should show new RNA isoforms in psoriatic lesional samples.

In the present study, we reanalyzed our previously published RNA-seq data (12) using transcript-based annotation to find alternatively spliced RNAs specific to psoriatic skin. We analyzed 173,446 different annotated RNA isoforms in the skin in relation to the existence of psoriatic lesion.

## Materials and Methods

### Patients and Controls

The Ethics Review Committee on Human Research of the University of Tartu approved the protocols and informed consent forms used in this study. All the study procedures conformed to the relevant regulatory standards. All the participants signed an informed consent form. The patients (*N* = 12) and control subjects (*N* = 12) were unrelated Caucasians living in Estonia. Patients from the Department of Dermatology of Tartu University Hospital with plaque psoriasis constituted the former group. Gender- and age-matched (±10 years) control subjects were recruited at the dermatologic outpatient clinic among patients with melanocytic nevi. These controls were free from inflammatory dermatoses and had no family history of psoriasis. The main characteristics of the psoriasis patients are presented in Table 1.

**Table 1:** **Characteristics of the psoriasis patients**.

Patient	Age in years	Gender	AoO (years)	PASI	Nail involvement	PsA
1	19	M	18 (0.5)	12.5	No	No
2	25	M	13 (12)	18.4	Yes	No
3	27	M	18 (9)	8.8	No	No
4	29	M	26 (3)	3.7	No	No
5	49	M	15 (34)	6.0	No	No
6	52	M	22 (30)	14.0	Yes	No
7	60	M	56 (4)	10.8	No	No
8	28	F	28 (0.5)	23.3	No	No
9	37	F	30 (7)	4.7	No	No
10	54	F	14 (40)	7.3	No	Yes
11	57	F	53 (4)	12.6	No	No
12	58	F	57 (1)	15.2	No	No

*AoO, age of onset; M, male; F, female; PASI, Psoriasis Area and Severity Index; PsA, psoriatic arthritis*.

### RNA Sequencing

The DNA fragment library was prepared using SOLiD System chemistry (Life Technologies Corp., Carlsbad, CA, USA). Also, 50 ng of total RNA was amplified using Ovation RNA-Seq System V2 (NuGen, Emeryville, CA, USA). Sequencing was performed using SOLiD 5,500 W. Also, 75 bp from the forward primer was sequenced, and on average 20 million reads per sample were received. Raw reads were mapped to the human genome hg19 reference, using CLC Genomic Workbench (Qiagen, Germany) software. The CLC Genomic Workbench identified a total of 173,446 transcripts, which were then used for downstream analysis.

### Quantitative Real-Time PCR

cDNA synthesis was performed on 250 ng of total RNA using a High Capacity RNA-to-cDNA Kit (Life Technologies Corp.). A QuantiTect Reverse Transcription Kit (Qiagen) was applied for cDNA synthesis in the case of g1 and s1 assays. Both cDNA synthesis kits were used according to the manufacturer’s protocol. cDNA was used as a template for TaqMan qRT-PCR using the 7900 Fast QRT-PCR System (Life Technologies Corp.). Two primers and a labeled probe were used to detect the mRNA expression level of the reference gene hypoxanthine phosphoribosyl-transferase-1 (HPRT-1; primer sequences available upon request). Expression levels of ETV3_3, S100A7A_2, IL36RN_4, and IL36G_3 were detected using TaqMan Gene Expression Assays (Life Technologies Corp.) Hs02786659_s1, Hs00752780_s1, Hs01104217_g1, and Hs00922858_m1, respectively. These assays were specific for the mentioned RNA isoforms and detected selectively only one isoform out of several variants. The assay specificity to detect alternative isoforms is illustrated in Figures S7–S10 in Supplementary Material. We selected genes for the qRT-PCR confirmation based on their statistical significance (ETV3 and S100A7A_2) from two different comparisons (LP–C and LP–NLP). IL36RN_4 and IL36G_3 were selected because these genes are recently identified targets in the pathogenesis of psoriasis, and it was important to analyze the expression of different isoforms. The gene expression levels from qRT-PCR were calculated relative to the reference gene HPRT-1 using the 2-ΔCT method. A paired *t*-test was used to evaluate any statistical significance between expressions.

### Statistical Analysis

We filtered out transcripts with low-expression level and did not use them in any further analysis. The EdgeR statistical package was used to analyze the non-normalized raw RNA isoform counts to find differential gene expressions. EdgeR performs model-based normalization, estimates dispersions, and applies a negative binomial model. EdgeR is a very flexible tool for RNA-seq data analysis for finding DEGs (16, 17). It fits a negative binomial model and then tests the procedures for determining differential expression.

We used a group comparison for samples of LP and NLP with C, and a paired comparison for LP and NLP. False discovery rate (FDR) adjustment was used for multiple testing corrections (18, 19). Our sample size of 12 patients provides sufficient power to find DEGs (20, 21).

## Results

### RNA Sequencing Results

We performed three comparisons: LP–C, NLP–C, and L–NLP. Of the 173,446 transcripts analyzed, 25,724 were expressed in all 36 samples at a ≥1 cpm (counts per million) level. In general, only one or two alternative isoforms of transcripts were expressed. In case of some genes, several different transcripts were expressed at the same time (e.g., SERPINB4).

**Figure 1:**
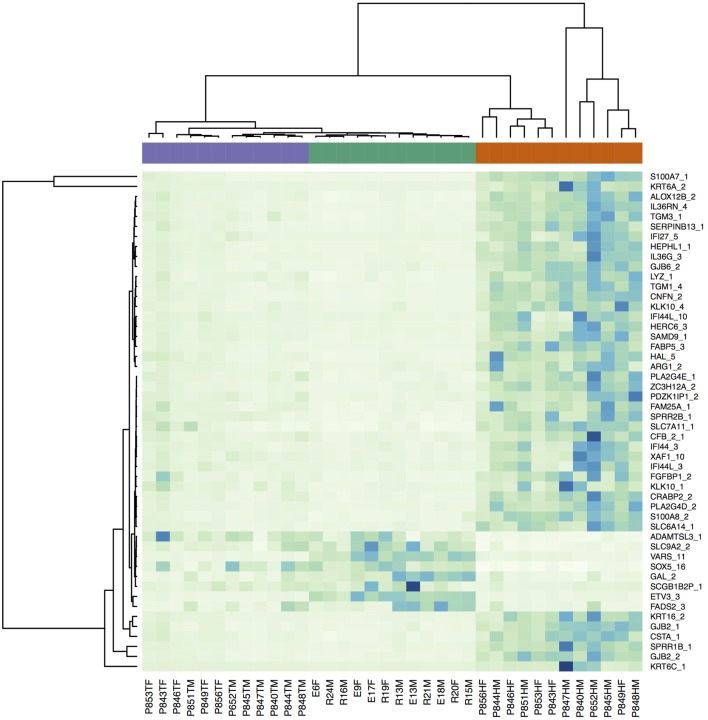
**Heatmap of the RNA-seq expression data for the 50 transcripts with the smallest FDR values (LP–C comparison) illustrating differences in transcriptional profiles**. The violet bar denotes non-lesional skin samples, the green bar controls, and the red bar lesional samples.

Figure S1 in Supplementary Material is a multidimensional scaling (MSD) plot that shows clear separation of the LP, NLP, and C samples. When differences between the LP and C samples were analyzed, 8,694 transcripts were differentially expressed at an FDR-corrected statistical significance level of 0.05. Table 2 provides the 25 most significant differentially expressed transcripts. The transcripts with the lowest FDR values also had the largest absolute logFC values, which indicate large expressional differences between samples. A heatmap (Figure 1) showed that the differences found should be considered consistent and characteristic of our study groups. The Volcano- and MA-plots on Figures S2 and S3 in Supplementary Material show reciprocal associations between logFC and FDR and gene expression level in our study sample.

**Table 2:** **Analysis of transcript isoforms in psoriasis, LP–C comparison, sorted by FDR value**.

Transcripts	logFC	logCPM	*p*-value	FDR	ENSEMBL ID	Gene name
ETV3_3	−4.12	7.41	8.29E−173	2.13E−168	ENST00000326786	Ets variant 3
NLK_6	−2.35	6.84	2.84E−90	3.65E−86	ENST00000407008	Nemo-like kinase
PLA2G4D_2	4.28	5.42	1.54E−66	1.32E−62	ENST00000290472	Phospholipase A2, group IVD (cytosolic)
TGM1_4	3.95	6.35	2.19E−54	1.41E−50	ENST00000206765	Transglutaminase 1
IL36RN_4	4.19	7.21	2.02E−51	1.04E−47	ENST00000393200	Interleukin 36 receptor antagonist
C0PG1_2	−2.68	4.28	4.32E−51	1.85E−47	ENST00000512034	Coatomer protein complex, subunit gamma 1
ALOX12B_2	2.96	7.24	2.64E−50	9.69E−47	ENST00000319144	Arachidonate 12-lipoxygenase, 12R type
SAMD9_1	2.94	6.16	5.35E−50	1.72E−46	ENST00000379958	Sterile alpha motif domain containing 9-like
STAT1_4	2.41	7.82	5,19E−49	1.48E−45	ENST00000361099	Signal transducer and activator of transcription 1.91 kDa
CRABP2_2	2.84	5.54	4.52E−46	1.16E−42	ENST00000368222	Cellular retinoic acid-binding protein 2
CNFN_2	3.71	6.67	1.90E−45	4.44E−42	ENST00000222032	Cornifelin
VARS_11	−3.50	4.58	2.07E−44	4.43E−41	ENST00000461328	Valyl-tRNA synthetase
KRT6A_2	4.50	11.56	2.67E−44	5.28E−41	ENST00000330722	Keratin 6A
CD300E_3	2.70	4.58	6.60E−44	1.21E−40	ENST00000392619	CD300e molecule
SOX5_16	−2.86	5.40	2.04E−42	3.49E−39	ENST00000546136	SRY (sex determining region Y)-box 5
SPTLC2_2	1.32	7.79	2.66E−42	4.27E−39	ENST00000216484	Serine palmitoyltransferase, long chain base subunit 2
TGM3_1	3.38	7.46	8.24E−42	1.25E−38	ENST00000381458	Transglutaminase 3
GLTP_5	2.02	7.68	8.90E−42	1.27E−38	ENST00000318348	Glycolipid transfer protein
FABP5_3	4.38	6.16	1.44E−41	1.95E−38	ENST00000481695	Fatty acid-binding protein 5 (psoriasis-associated)
LYZ_1	3.05	6.50	4.50E−41	5.79E−38	ENST00000261267	Asparagine synthetase (glutamine-hydrolyzing)
IL36G_3	5.43	6.53	5.41E−40	6.62E−37	ENST00000259205	Interleukin 36, gamma
HERC6_3	4.31	5.99	1.56E−39	1.83E−36	ENST00000264346	HECT and RLD domain containing E3 ubiquitin protein ligase family member 6
CLSTN2_1	−2.05	5.74	1.51E−38	1.68E−35	ENST00000458420	Calsyntenin 2
PGAM1_1	2.04	6.40	7.24E−38	7.76E−35	ENST00000334828	Phosphoglycerate mutase 1 (brain)
KRT16_2	4.73	8.10	1.27E−37	1.31E−34	ENST00000301653	Keratin 16

*Differentially expressed transcripts in psoriatic lesions compared to control skin (LP–C). LogFC is fold-change differences on the log2 scale and describes how many times gene expression differs between groups. Positive values indicate gene upregulation in psoriasis patients. Log2 counts per million (LogCPM) is the average gene expression signal of all samples. FDR is a genome-wide corrected *p*-value*.

**Figure 2:**
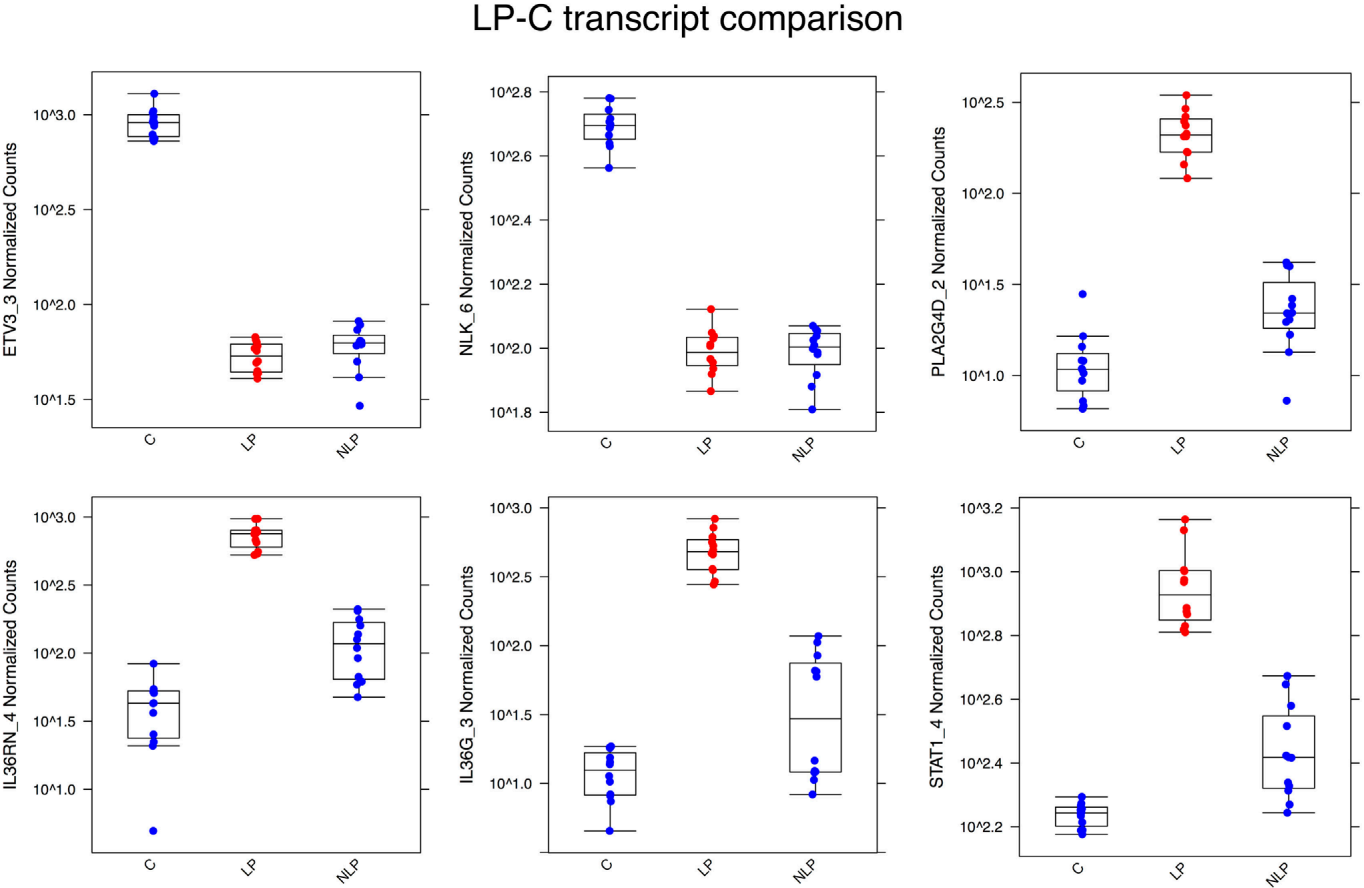
**Box plots illustrating some of the most differentially expressed transcripts between the LP and C samples**. ETV3_3 and NLK_6 were highly expressed in control skin but downregulated in lesional and non-lesional skin. The other genes in the figures were all upregulated in the psoriatic samples, but less so in non-lesional skin. Abbreviations in the *X*-axis are LP, lesional, NLP, non-lesional, and C, control.

The most significantly downregulated transcript in the LP sample was ETV3_3, followed by NLK_6, a nemo-like kinase. Most of the transcripts that were significantly upregulated in the LP sample are known to be related to the activation of immune functions. PLA2G4D, TGM1, and IL36RN are all described to be involved in the psoriasis (11, 12). IL36RN is quite recently identified to have substantial role in different inflammatory skin diseases (22). A box plot of the differential expression levels of selected transcripts is illustrated in Figure 2.

When we compared NLP skin samples with C samples, the most significantly downregulated transcripts in NLP skin (like in LP skin) were ETV3_3 and NLK_6 (see Table S2 in Supplementary Material). Expression levels of the 50 most differentially expressed transcripts (NLP–C) are illustrated in the heatmap Figure S4 in Supplementary Material.

And finally, we performed a pairwise comparison of lesional (LP) and non-lesional (NLP) samples to identify the transcripts that are involved in generation of active lesion (Table S3 in Supplementary Material). The most upregulated (the highest logFC) transcripts of LP were S100A7A_2, S100A8_2, S100A7_1, and KLK10_4. S100A7A_2 corresponds to the long isoform (previously described as S100A15_L) of S100A15 or S100A7A that was identified long time ago (23). This long isoform is identified to be specific for lesional psoriatic skin, and our present results support this finding (24).

Expression profiles of the 100 most significantly different genes are illustrated using a heatmap (see Figure S5 in Supplementary Material). The box plot of the expression levels of selected genes from LP–NLP comparison is illustrated in Figure S6 in Supplementary Material. In addition to the transcripts found also in other comparisons (IL36RN), specific transcripts of S100A8, MPZL2, KLK10, and EHF genes were identified in relation to active lesion in the skin. In case of EHF, the transcript EHF_12 (ENST00000257831) corresponds to the longest RNA variant, but it is not coding the longest protein of all 100 EHF variants. Interestingly, these transcripts interact with the psoriasis response element motif and are necessary in the development of psoriasis (25). EHF is an “ets homologous factor” and distantly related to the ETV3 gene that was downregulated in the LP and NLP skin samples compared to the controls.

### Validation of RNA-Seq Results with Quantitative Real-Time PCR

**Figure 3:**
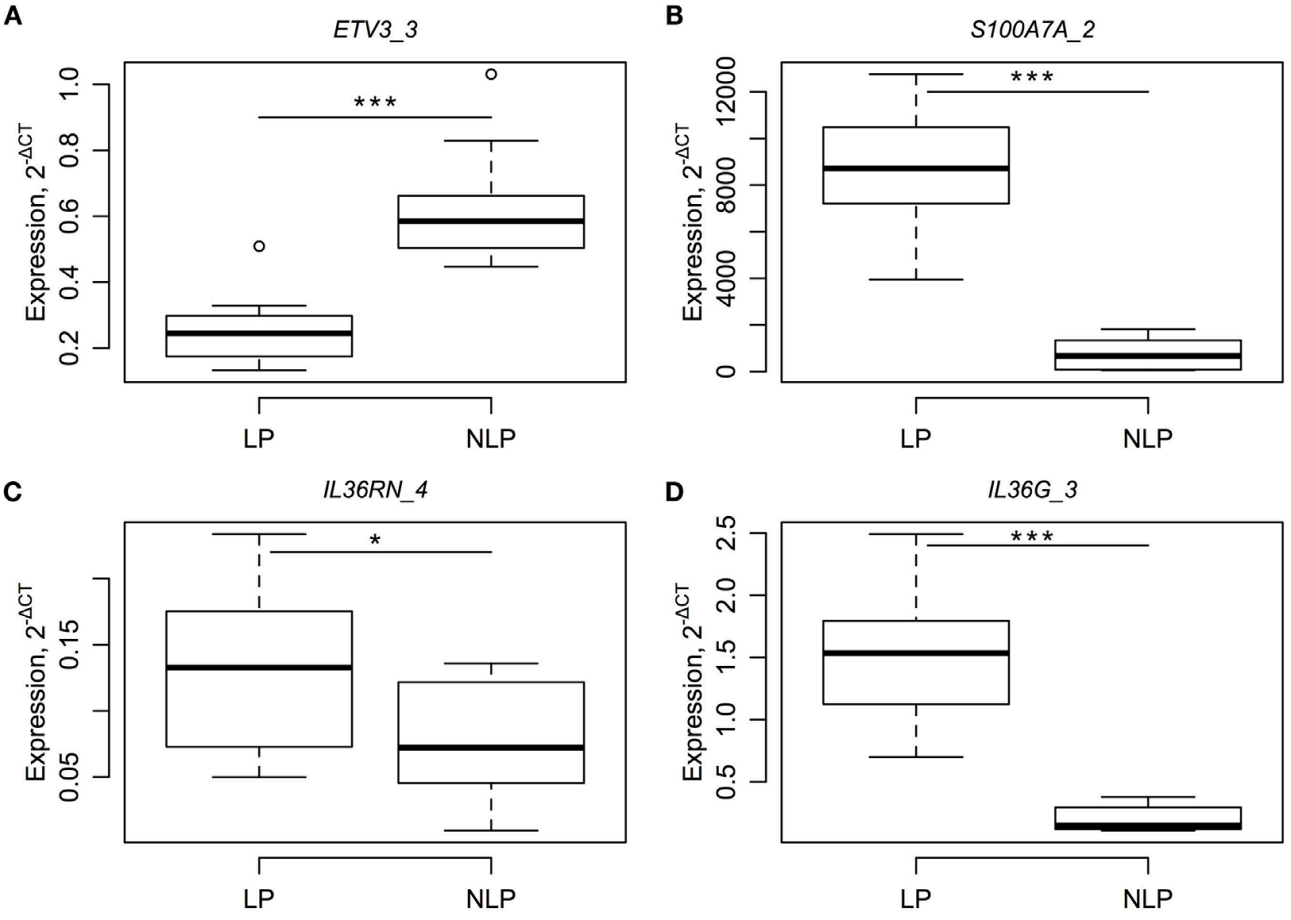
**RT-PCR results for four different RNA isoforms (A–D) and a comparison between paired LP and NLP samples**. RNA levels of individual genes (**A–D** are ETV3_3, S100A7A_2, IL36RN_4 and IL36G_3) are expressed relative level to the housekeeper gene. Statistical significance for the paired *t*-tests is designated as *, a *p*-value less than 0.05 and ***, a *p*-value less than 0.001.

In order to confirm the RNA-seq results, we measured the levels of ETV3_3, S100A7A_2, IL36RN_4, and IL36G_3 using real-time PCR for the paired LP–NLP samples. All RT-PCR assays were selected so that they only detected singe specific RNA isoforms and no other RNA isoforms encoded by the same gene. RT-PCR analysis confirmed significant differences in the expression of gene isoforms between the LP and NLP samples (Figure 3).

## Discussion

In the present study, we present the differences in alternative splicing for lesional (LP) and non-lesional (NLP) psoriasis skin samples compared to control (C) skin samples. We reanalyzed our published data by applying transcript-based annotation (12). This annotation enabled analysis of 173,446 transcripts in the human genome to find differentially regulated RNA isoforms.

In one previous study, exon-specific differential expression in psoriasis samples was analyzed (11). The authors found 343 exons differentially used between lesional and control skin samples (11). Unfortunately, the results were presented only in a descriptive table with no formal comparison of lesional to NLP skin. Comparison of our results with published Li et al. (11) results could therefore be only limited. Mentioned previous study indicated differential exon usage for IL1F5 (IL36RN) and IL1F7 (IL37) genes (11). In present study, we found that the same genes and their specific RNA variants are differentially expressed in psoriasis samples. Therefore, in general, our results fit well with the findings of the Li et al. Our results even extend their findings, and we identified novel RNA isoforms that are differentially expressed. Some discrepancies between Li et al. and our findings come form different approach. Li et al. used differential exon usage analysis that is implemented in the DEXseq package. This analysis detects the exons that are differentially expressed but is not able to find the exact specific RNA isoforms. As a result, they found that 343 exons from 292 genes were differentially expressed (11). Our approach resulted in more than 8,000 differentially expressed transcripts between LP and C skin. In order to verify our findings, we downloaded the original data used in the study of Li et al. and applied our approach. Indeed, our approach found more than 10,000 transcripts differentially expressed between psoriasis and control samples. Moreover, we confirmed differential expression for the RNA isoforms for ETV3, IL36G, PLA2G4D, and S100A7A with RT-PCR. Taken together, our approach is applicable for different datasets and gives consistent findings in our and in the previously published datasets.

The most significant differentially expressed transcript between LP and C was ETV3_3 (ENST00000326786, see Figure S7 in Supplementary Material ETV3-001). Interestingly, the most significant differentially expressed transcript is not the longest. It is 1,539 bp in length and encodes 143 aa proteins. The ETV3 has two additional transcripts, ENST00000368192 and ENST00000460850. ENST00000368192 is 5,254 bp long and encodes protein with 512 aa. ENST00000460850 was the shortest (696 bp in length) and is a non-coding transcript. Both ENST00000368192 and ENST00000460850 were expressed in the skin samples, but their expression did not differ between the experimental groups. Therefore, ETV3_3 seems to be specific to psoriasis, and its downregulation in LP samples was remarkable (logFC −4.06). In the case of NLK_6 (ENST00000407008), it is the one splicing variant out of six existing variants of NLK gene and encodes the 527 aa protein. In addition to NLK_6, only NLK_2 (ENST00000582037) of all the NLK isoforms was differentially expressed (FDR value below 0.05). NLK_2 encodes the 181 aa protein of the NLK gene. Both ETV3_3 and NLK_6 were downregulated in the LP sample. The ETV3 gene functions as a component of the IL-10-regulated pathway that represses inflammatory response (26). IL-10 and the IL-10 family of cytokines are involved in genetic susceptibility to psoriasis vulgaris and palmoplantar pustulosis (27, 28). Therefore, ETV3 seems to be a good candidate for regulating psoriasis inflammation. ETV3 was initially discovered as an ETS family repressor of gene expression in macrophage development (29). The second most significantly downregulated transcript in the LP sample was NLK_6. NLK is also related to immune suppression, and it negatively regulates NF-kappaB (30). However, our RT-PCR experiment did not verify the differential expression of NLK_6 isoform, but this transcript still remains interesting candidate.

A comparison between LP and NLP samples should describe the lesion-specific changes induced by the active inflammation in the skin. Two splicing variants of S100A8 (ENST00000368733 and ENST00000368732), one variant of S100A7A (ENST00000368729), and one variant of S100A7 (ENST00000368723) were significantly upregulated in lesional skin with very high logFC values. In case of these genes, out of two or three existing transcripts for these genes were specifically changed. S100A7A_2 corresponds to the long isoform (previously described as S100A15_L) of S100A15 or S100A7A gene that was cloned and identified in an earlier study (23). Interestingly, this long isoform was found to be specific to lesional psoriatic skin and therefore supports our finding of lesional – specificity (24). Moreover, TNF-α, IFN-γ, and IL-1β have shown to stimulate only long isoform (24). We found that S100A15_L (S100A7A_2) is slightly upregulated in non-lesional skin (compared to control) and is highly upregulated in lesional skin. Skin does not normally express the S100A7A long isoform at all and only slightly expresses the S100A7A short isoform (23). Therefore, S100A7A is perfect example for the transcript-specific regulation of inflammation and disease progression. In addition, S100A8 is also a well-described and well-known psoriasis-inducing gene (31). There is evidence that S100A8 binds to unsaturated fatty acids (32). Supportive to this interaction was up-regulation of the fatty acid-binding protein FABP5 in our samples. FABP5 gene has also been related to psoriasis (33). Finally, the IL36RN_4 splicing variant of the IL36RN gene was similarly upregulated in the lesional sample. Therefore, our findings from the lesional to non-lesional skin sample comparison coincide with several previous studies. All the splicing variants and respective detection assays for RT-PCR are illustrated in the Figures S7–S10 in Supplementary Material. As seen in the figure, assays were specific for the splicing variants.

In conclusion, our approach in analyzing the specific pattern of splicing variants produced some new candidates for the pathogenesis of psoriasis and indicated transcript-specific regulation. Downregulation of ETV3 in psoriatic skin (lesional and non-lesional) indicates a suppression of anti-inflammatory signals for psoriasis. We confirmed the finding of a previous study by detecting the up-regulation of S100A7A long isoform in lesional skin (24). RNA-seq analysis, where specific transcript splicing variants are analyzed, provides substantial additional information compared to regular gene-based analysis where signals from different transcripts are combined.

## Author Contributions

SK – conceived study, data analysis, and manuscript writing. MK – clinical sample collection, clinical expertise, and validation. ER – RNA-seq analysis, library preparation, and sequencing. EP – RNA-seq analysis. KA – clinical expert. HS – clinical expert and manuscript writing. GK – data analysis. KK – clinical expert, lead of the clinical group, and conceived the study.

## Conflict of Interest Statement

The authors declare that the research was conducted in the absence of any commercial or financial relationships that could be construed as a potential conflict of interest.

## Supplementary Material

Figure S1 **Multidimensional scaling plot of the original data indicating a clear separation of the experimental groups non-lesional psoriatic skin (NLP) and lesional psoriatic skin (LP) with controls (C)**.

Figure S2 **Volcano plot shows the relationships between *p*-values (−log 10) and fold-change differences (log 2)**. The transcripts from the LP–C comparison are illustrated.

Figure S3 **MA-plot of the transcripts used for the LP–C comparison**. Transcripts with larger expressional levels had larger fold-change differences.

Figure S4 **Heatmap showing the expression of 50 of the most differentially expressed transcripts between the non-lesional and control samples**. Data from all three groups are illustrated, and it was evident that some genes overlap with lesional samples and some with control samples. Transcripts common in both LP and NLP samples indicate that even in the non-lesional skin of psoriatic patients, the pathogenetic transcripts are active. The violet bar denotes controls, the green bar lesional skin samples, and the red bar non-lesional samples.

Figure S5 **Heatmap showing the expression of 100 of the most differentially expressed transcripts between the non-lesional and lesional samples**. The green bar denotes non-lesional skin samples and the red bar lesional samples.

Figure S6 **Box plots illustrating some of the most differentially expressed transcripts between the LP and NLP samples**. Abbreviations in the *X*-axis are LP, lesional; NLP, non-lesional.

Figure S7 **This figure illustrates the different transcripts for the ETV3 gene and the position of the TaqMan assay (red box)**. ETV3_001 was the transcript found to be specifically downregulated in the case of psoriasis.

Figure S8 **This figure illustrates the different transcripts for the S100A7A gene and the position of the TaqMan assay (red box)**. S100A7A 001 was the transcript found to be specifically downregulated in the case of psoriasis.

Figure S9 **This figure illustrates the different transcripts for the IL36RN gene and the position of the TaqMan assay (red box)**. IL36RN _001 was the transcript found to be specifically downregulated in the case of psoriasis.

Figure S10 **This figure illustrates the different transcripts for the IL36G gene and the position of the TaqMan assay (red box)**. IL36G _001 was the transcript found to be specifically downregulated in the case of psoriasis.

Table S1 **Differentially expressed transcripts in lesional skin compared to controls (LP–C)**. Data are sorted descending in terms of absolute logFC values.

Table S2 **Differentially expressed transcripts in non-lesional skin compared to controls (NLP–C)**. Data are sorted descending in terms of absolute logFC values.

Table S3 **Differentially expressed transcripts in lesional skin compared to non-lesional skin (LP–NLP)**.
