# Supplementary figures and images for: Psoriasis-Specific RNA Isoforms Identified by RNA-Seq Analysis of 173,446 Transcripts
Source: Front Med (Lausanne). 2016 Oct 7;3:46. doi: 10.3389/fmed.2016.00046 (PMC5053979; doi:10.3389/fmed.2016.00046)

# MDS Plot of psoriasis RNAseq

Figure 1S

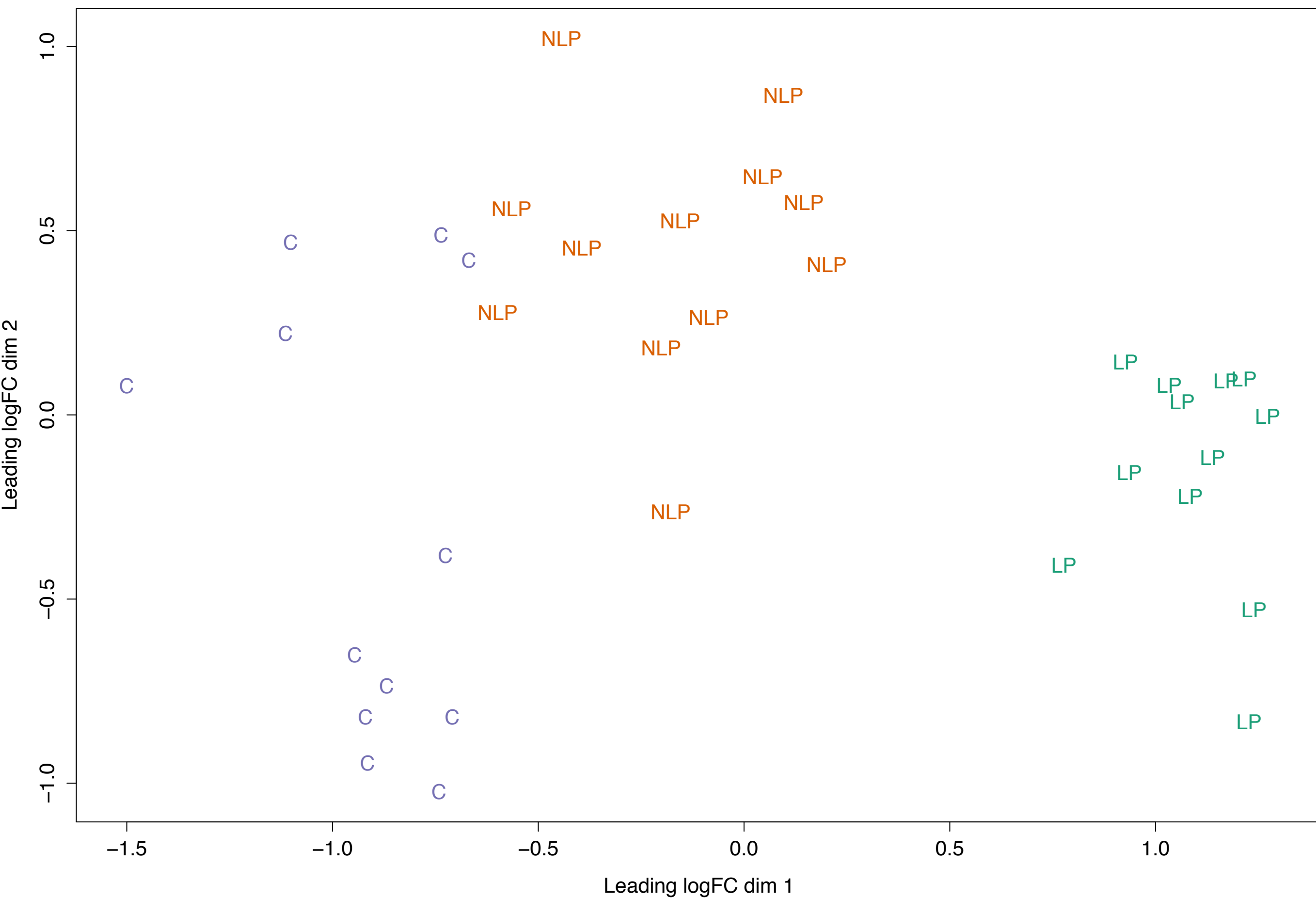

Supplement: Figure S1 — Multidimensional scaling plot of the original data indicating a clear separation of the experimental groups non-lesional psoriatic skin (NLP) and lesional psoriatic skin (LP) with controls (C). [file Image_1.PDF]

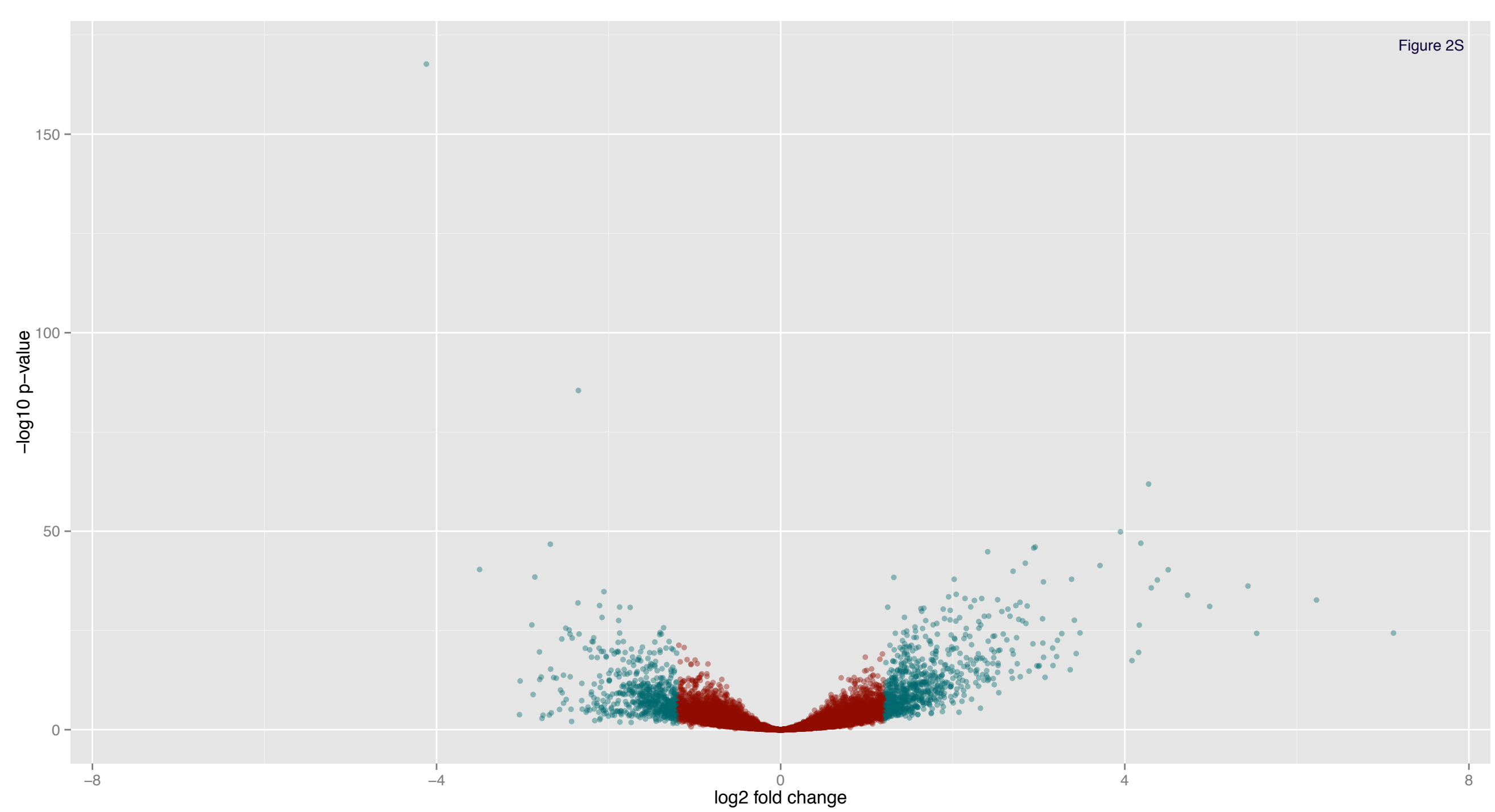

Supplement: Figure S2 — Volcano plot shows the relationships between p-values (−log 10) and fold-change differences (log 2). The transcripts from the LP–C comparison are illustrated. [file Image_2.PDF]

## MA Plot LP-C edgeR

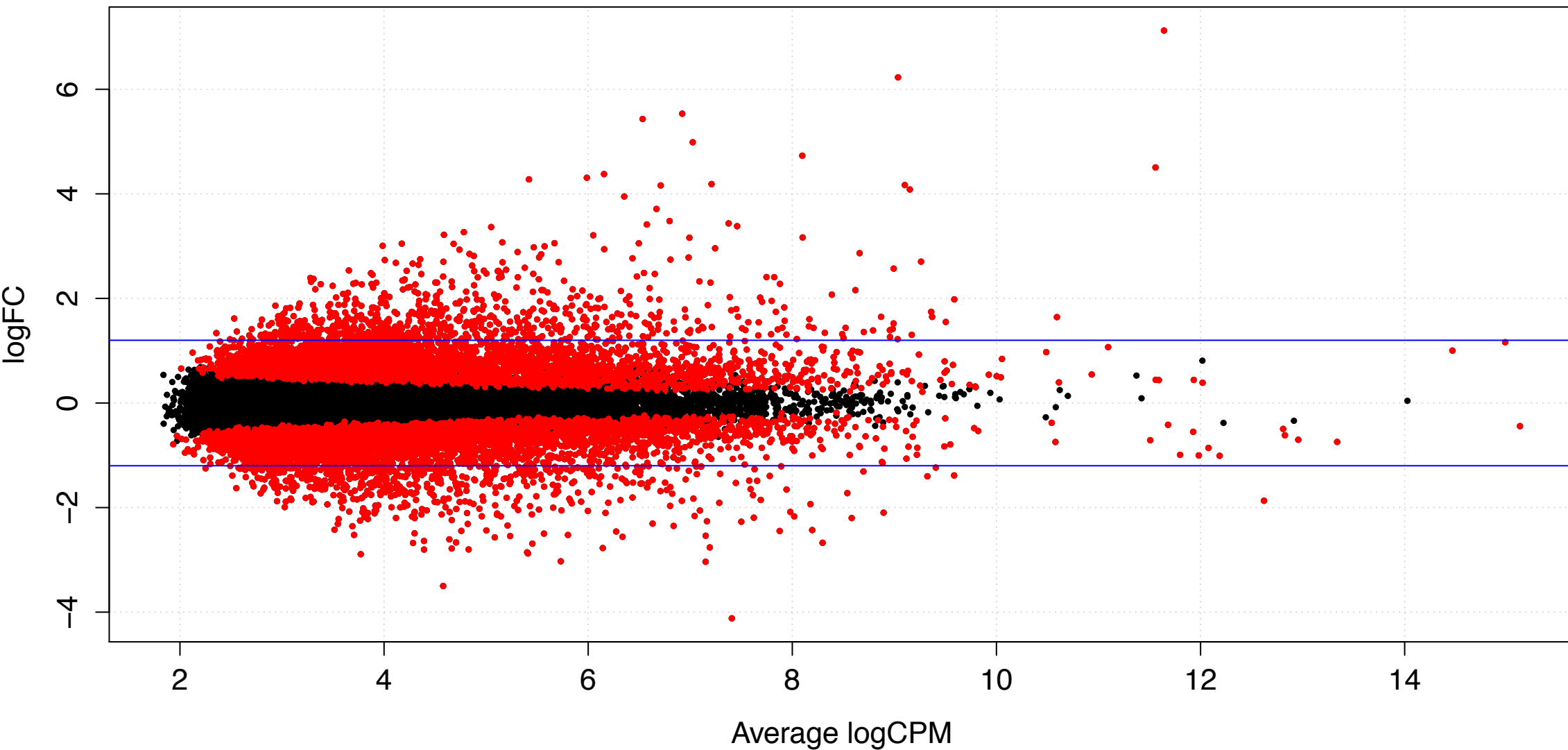

Supplement: Figure S3 — MA-plot of the transcripts used for the LP–C comparison. Transcripts with larger expressional levels had larger fold-change differences. [file Image_3.PDF]

Figure 4S

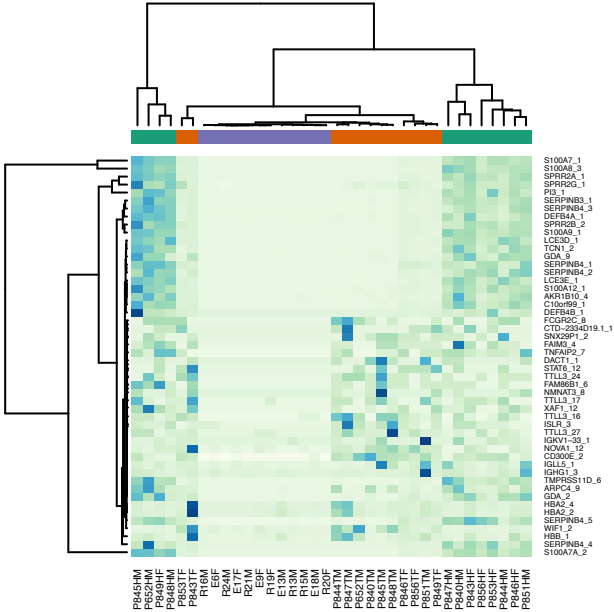

Supplement: Figure S4 — Heatmap showing the expression of 50 of the most differentially expressed transcripts between the non-lesional and control samples. Data from all three groups are illustrated, and it was evident that some genes overlap with lesional samples and some with control samples. Transcripts common in both LP and NLP samples indicate that even in the non-lesional skin of psoriatic patients, the pathogenetic transcripts are active. The violet bar denotes controls, the green bar lesional skin samples, and the red bar non-lesional samples. [file Image_4.PDF]

Figure 5S

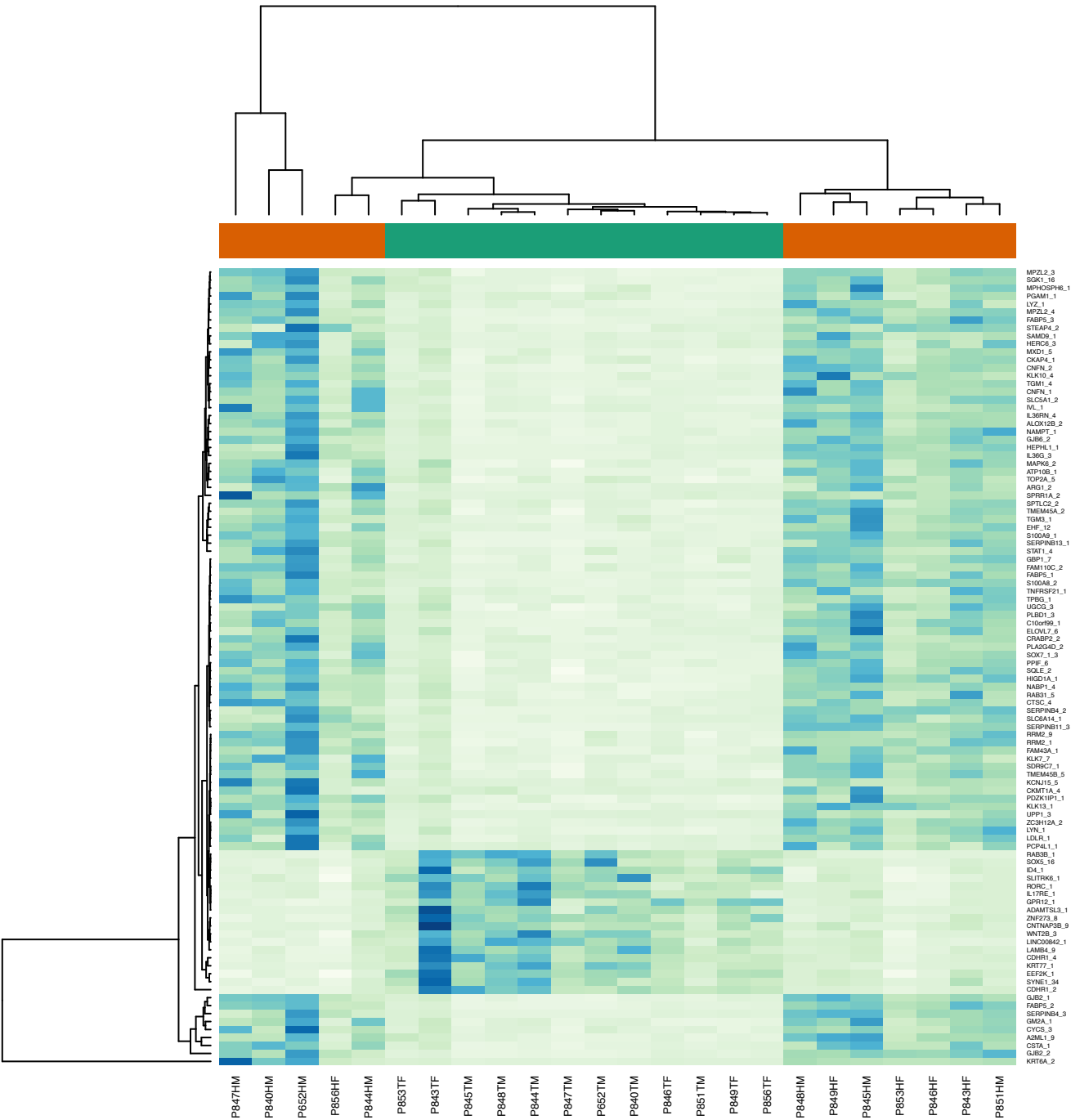

Supplement: Figure S5 — Heatmap showing the expression of 100 of the most differentially expressed transcripts between the non-lesional and lesional samples. The green bar denotes non-lesional skin samples and the red bar lesional samples. [file Image_5.PDF]

Figure 6S

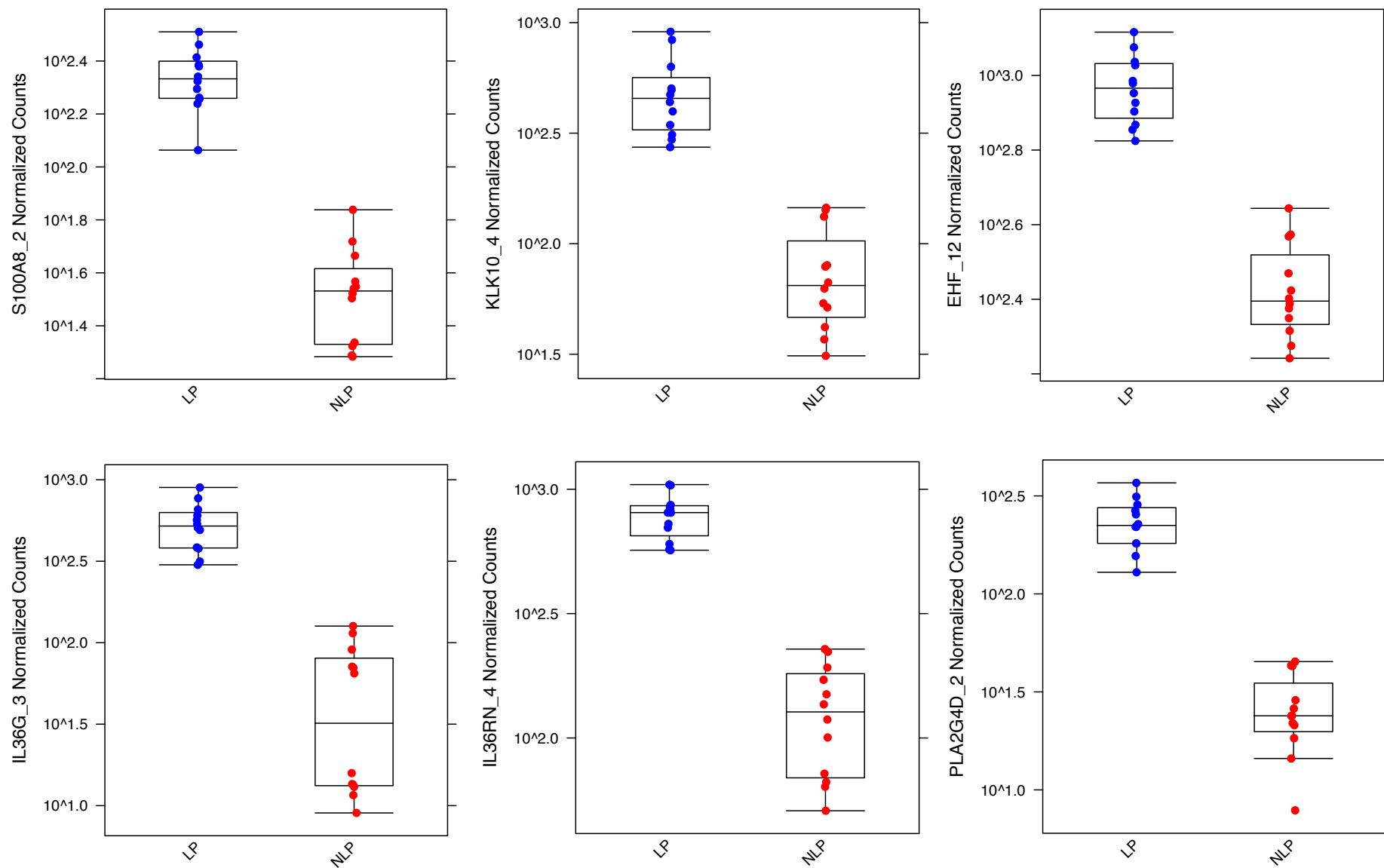

Supplement: Figure S6 — Box plots illustrating some of the most differentially expressed transcripts between the LP and NLP samples. Abbreviations in the X-axis are LP, lesional; NLP, non-lesional. [file Image_6.PDF]

Figure 7S

NCBI Build 37.1 Human Chr.1: Hs02786659\_s1

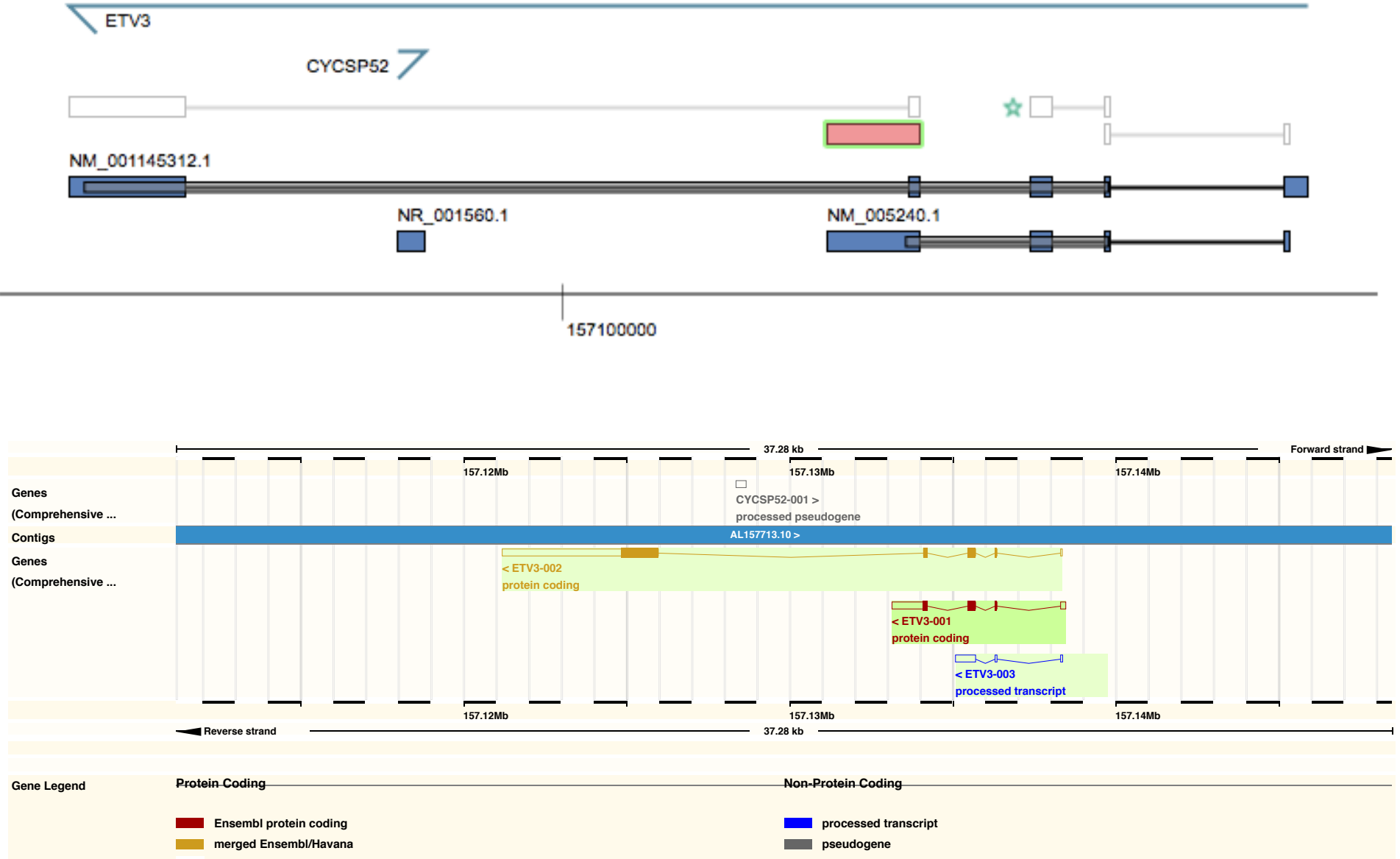

Supplement: Figure S7 — This figure illustrates the different transcripts for the ETV3 gene and the position of the TaqMan assay (red box). ETV3_001 was the transcript found to be specifically downregulated in the case of psoriasis. [file Image_7.PDF]

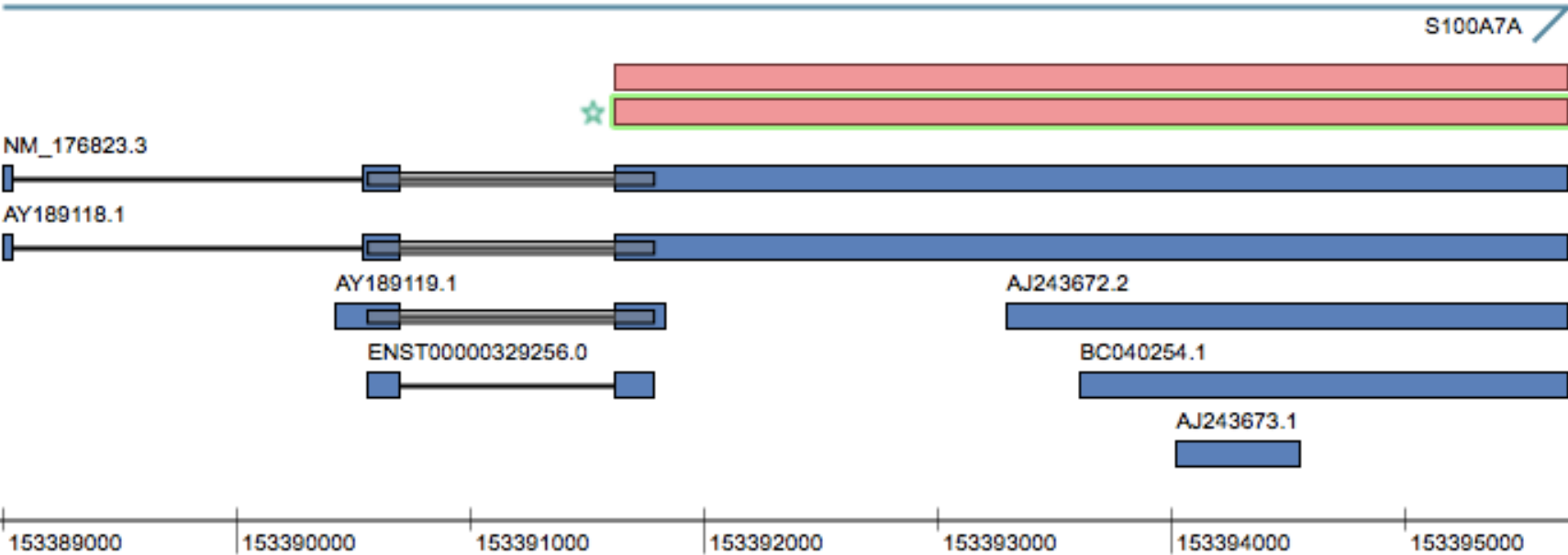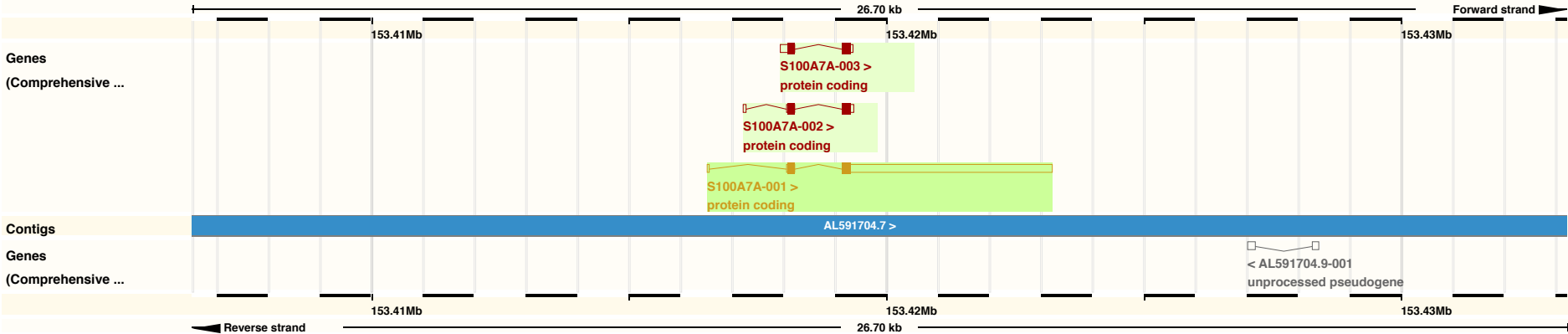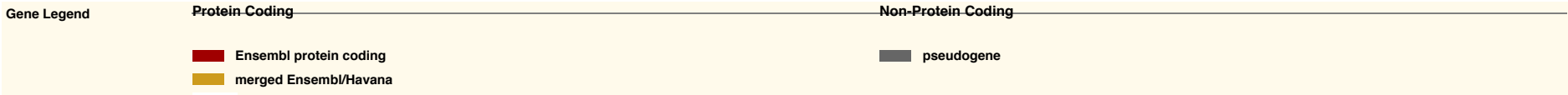

Supplement: Figure S8 — This figure illustrates the different transcripts for the S100A7A gene and the position of the TaqMan assay (red box). S100A7A 001 was the transcript found to be specifically downregulated in the case of psoriasis. [file Image_8.PDF]

Figure 9S

NCBI Build 37.1 Human Chr.2: Hs01104217\_g1

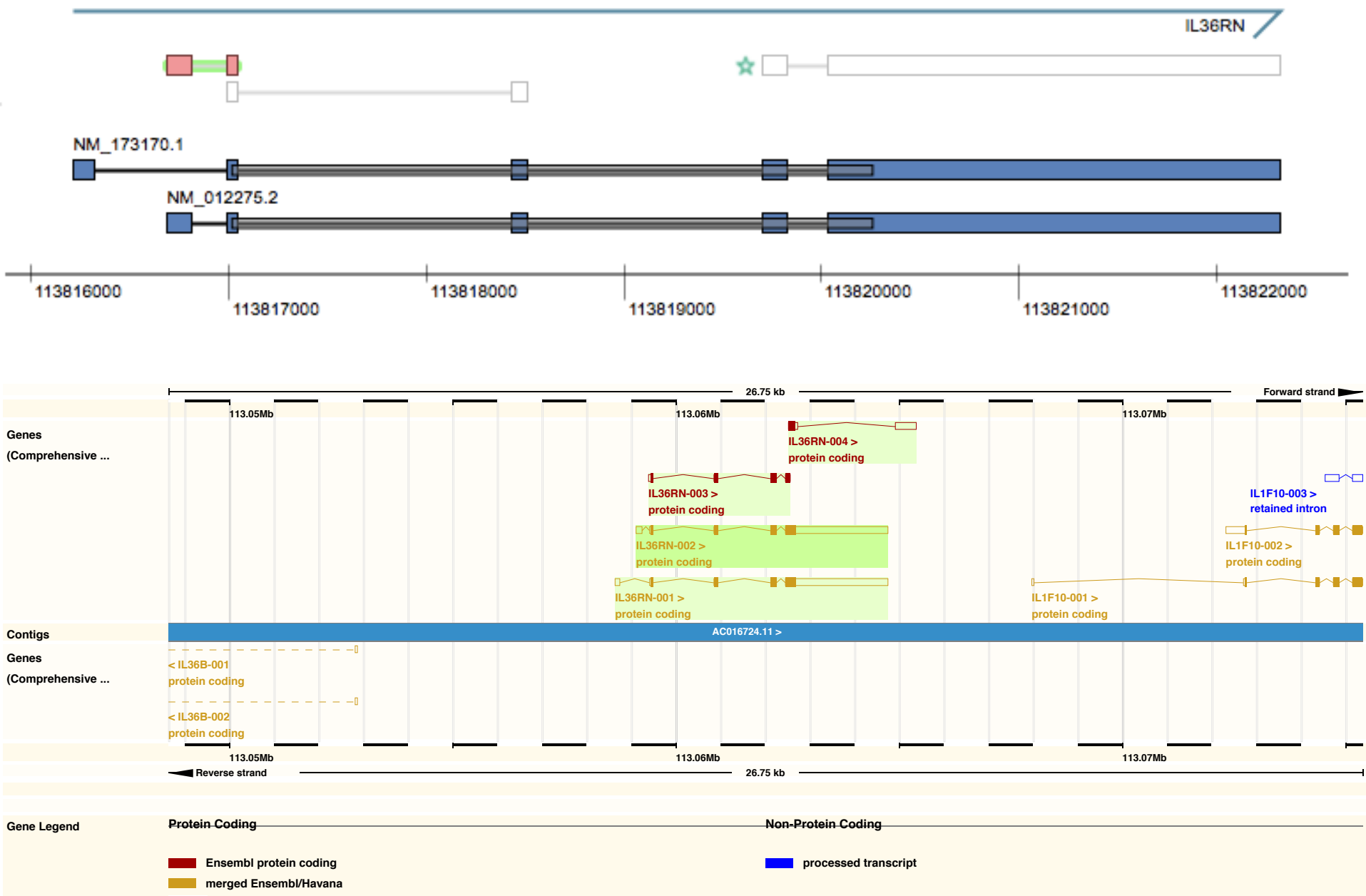

Supplement: Figure S9 — This figure illustrates the different transcripts for the IL36RN gene and the position of the TaqMan assay (red box). IL36RN _001 was the transcript found to be specifically downregulated in the case of psoriasis. [file Image_9.PDF]

Figure 10S

NCBI Build 37.1 Human Chr.2: Hs00922858\_m1

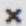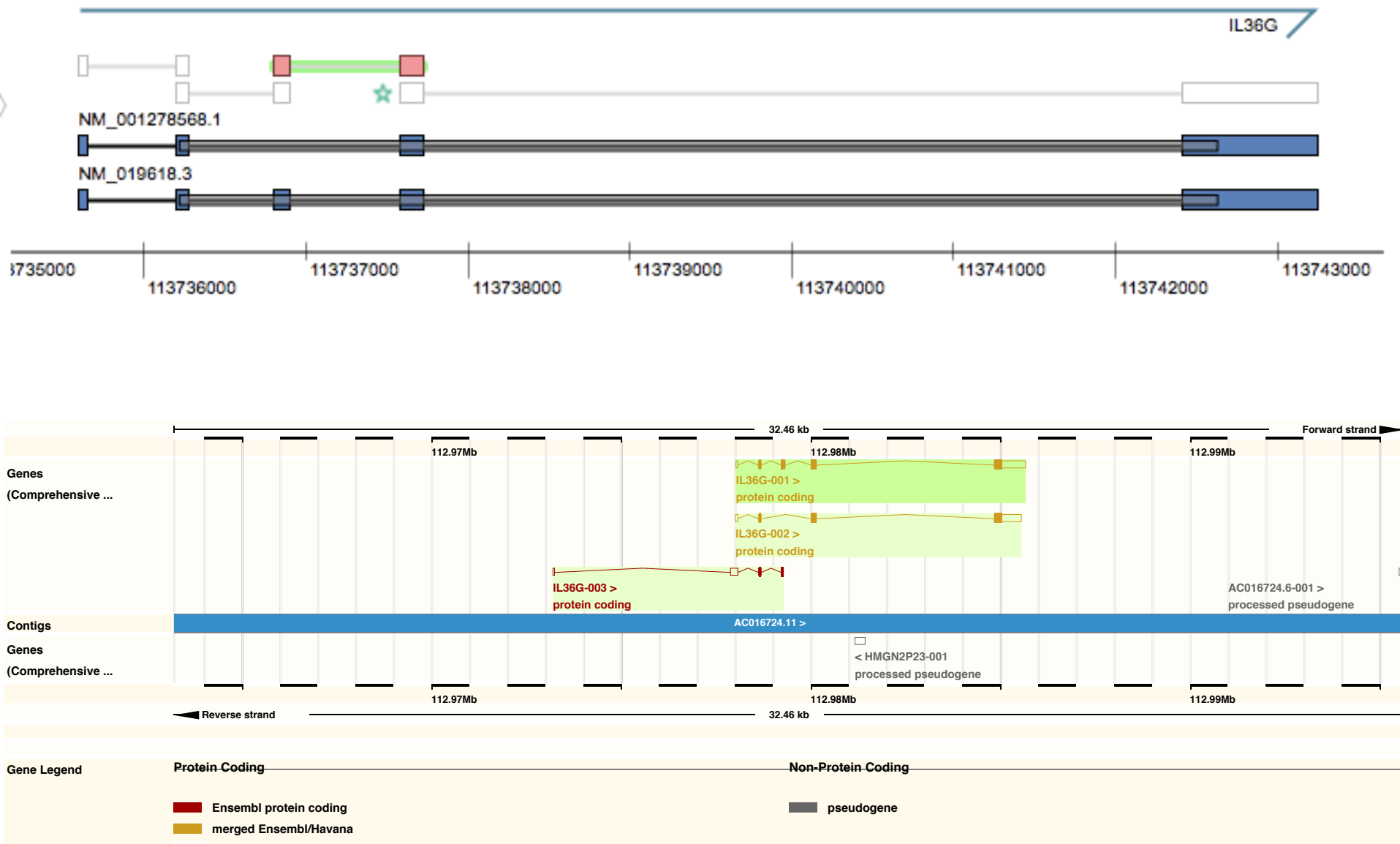

Supplement: Figure S10 — This figure illustrates the different transcripts for the IL36G gene and the position of the TaqMan assay (red box). IL36G _001 was the transcript found to be specifically downregulated in the case of psoriasis. [file Image_10.PDF]
