# Supplementary material for: Psoriasis-Specific RNA Isoforms Identified by RNA-Seq Analysis of 173,446 Transcripts
Source: Front Med (Lausanne). 2016 Oct 7;3:46. doi: 10.3389/fmed.2016.00046 (PMC5053979; doi:10.3389/fmed.2016.00046)
Supplement: Table S1 — Differentially expressed transcripts in lesional skin compared to controls (LP–C). Data are sorted descending in terms of absolute logFC values. [file Table_1.PDF]

Table 1S. Analysis of transcript isoforms in psoriasis, LP-C comparison, sorted by logFC.

Transcripts	logFC	logCPM	PValue	FDR	gene name
S100A7_1	7,123395	11,641784	1,45E-27	4,50E-25	S100 calcium binding protein A7
KRT6C_1	6,228201	9,036259	2,54E-36	2,17E-33	keratin 6C
HEPHL1_1	5,533319	6,921536	1,78E-27	5,31E-25	hephaestin-like 1
IL36G_3	5,431648	6,533205	5,41E-40	6,62E-37	interleukin 36, gamma
IFI27_5	4,987286	7,02414	1,29E-34	8,97E-32	interferon, alpha-inducible protein 27
KRT16_2	4,729997	8,097777	1,27E-37	1,31E-34	keratin 16
KRT6A_2	4,504749	11,558661	2,67E-44	5,28E-41	keratin 6A
FABP5_3	4,378333	6,155371	1,44E-41	1,95E-38	fatty acid binding protein 5 (psoriasis-associated)
HERC6_3	4,308217	5,986902	1,56E-39	1,83E-36	HECT and RLD domain containing E3 ubiquitin protein ligase, member 6
PLA2G4D_2	4,277096	5,419597	1,54E-66	1,32E-62	phospholipase A2, group IVD
IL36RN_4	4,186447	7,208713	2,02E-51	1,04E-47	interleukin 36 receptor antagonist
GJB2_2	4,16841	9,10238	1,22E-29	4,50E-27	gap junction protein, beta 2, 26kDa
ARG1_2	4,160585	6,71047	2,31E-22	3,42E-20	arginase 1
ETV3_3	-4,118337	7,407215	8,29E-173	2,13E-168	ets variant 3
SPRR1B_1	4,083639	9,151773	3,36E-20	3,85E-18	small proline-rich protein 1B
TGM1_4	3,949811	6,35337	2,19E-54	1,41E-50	transglutaminase 1
CNFN_2	3,711599	6,669105	1,90E-45	4,44E-42	cornifelin
VARS_11	-3,499852	4,579038	2,07E-44	4,43E-41	valyl-tRNA synthetase
HAL_5	3,479674	6,797562	1,30E-27	4,09E-25	histidine ammonia-lyase
SERPINB13_1	3,435087	7,375723	4,71E-22	6,66E-20	serpin peptidase inhibitor, clade B (ovalbumin), member 13
KLK10_4	3,414222	6,574687	6,05E-31	2,64E-28	kallikrein-related peptidase 10
TGM3_1	3,381818	7,459051	8,24E-42	1,25E-38	transglutaminase 3
IFI44L_3	3,365207	5,049822	9,15E-18	7,98E-16	interferon-induced protein 44-like
PDZK1IP1_2	3,267865	4,781973	2,01E-27	5,93E-25	PDZK1 interacting protein 1
XAF1_10	3,216867	4,586613	1,33E-25	3,07E-23	XIAP associated factor 1
IFI44L_10	3,207916	6,049747	2,84E-21	3,78E-19	interferon-induced protein 44-like
GJB2_1	3,165826	8,101106	6,99E-19	6,81E-17	gap junction protein, beta 2, 26kDa

GJB6_2	3,161411	6,99165	1,50E-23	2,74E-21	gap junction protein, beta 6, 30kDa
SLC7A11_1	3,073268	5,158925	9,59E-16	6,34E-14	solute carrier family 7, member 11
SLC6A14_1	3,056188	5,670002	4,40E-21	5,71E-19	solute carrier family 7, member 14
