# Supplementary material for: Psoriasis-Specific RNA Isoforms Identified by RNA-Seq Analysis of 173,446 Transcripts
Source: Front Med (Lausanne). 2016 Oct 7;3:46. doi: 10.3389/fmed.2016.00046 (PMC5053979; doi:10.3389/fmed.2016.00046)
Supplement: Table S2 — Differentially expressed transcripts in non-lesional skin compared to controls (NLP–C). Data are sorted descending in terms of absolute logFC values. [file Table_2.PDF]

Table 2S. Analysis of transcript isoforms in psoriasis, NLP-C comparison, sorted by logFC.

Transcriots	logFC	logCPM	PValue	FDR	gene name
ETV3_3	-3,94	7,41	4,78E-161	1,23E-156	ets variant 3
VARS_11	-3,74	4,58	8,04E-50	5,17E-46	valyl-tRNA synthetase
S100A7_1	3,71	11,64	3,17E-11	1,66E-08	S100 calcium binding protein A7
COPG1_2	-2,75	4,28	2,27E-53	1,95E-49	coatomer protein complex, subunit gamma 1
VARS_3	-2,64	3,51	8,47E-29	2,72E-25	valyl-tRNA synthetase
CHD2_3	-2,60	5,57	5,20E-31	1,91E-27	chromodomain helicase DNA binding protein 2
ALOX15B_2	-2,57	4,71	1,27E-05	8,08E-04	arachidonate 15-lipoxygenase, type B
CD300E_3	2,50	4,58	2,83E-37	1,21E-33	CD300e molecule
KRT6C_1	2,42	9,04	7,85E-09	1,96E-06	keratin 6C
ALOX15B_3	-2,37	5,08	3,58E-06	3,05E-04	arachidonate 15-lipoxygenase, type B
NLK_6	-2,35	6,84	2,03E-90	2,61E-86	nemo-like kinase
KDM6B_2	-2,35	5,80	1,42E-14	1,14E-11	lysine (K)-specific demethylase 6B
FAR2P2_5	-2,29	5,24	1,02E-21	1,88E-18	fatty acyl CoA reductase 2 pseudogene 2
GAL_2	-2,29	5,73	1,63E-09	5,38E-07	galanin
HEPHL1_1	2,27	6,92	3,88E-07	5,23E-05	hephaestin-like 1
IFI27_5	2,25	7,02	8,04E-10	2,87E-07	interferon, alpha-inducible protein 27
MMP2_1	2,17	7,75	1,51E-12	1,05E-09	matrix metallopeptidase 2
TNXB_6	2,15	3,88	2,56E-08	5,16E-06	tenascin XB
INSIG1_4	-2,14	4,66	4,42E-07	5,77E-05	insulin induced gene 1
RP11-867G23.3_1	-2,14	4,66	1,34E-10	5,85E-08	RP11-867G23.3, lincRNA
DPP4_2	2,13	5,38	3,99E-19	4,89E-16	dipeptidyl-peptidase 4
CPM_6	-2,12	4,37	5,13E-21	8,25E-18	carboxypeptidase M
ST6GAL2_5	-2,11	7,50	9,69E-21	1,47E-17	ST6 beta-galactosamide alpha-2,6-sialyltranferase 2
ELOVL5_8	-2,09	5,66	2,64E-05	1,41E-03	ELOVL fatty acid elongase 5
ELOVL5_4	-2,09	5,45	1,96E-09	6,31E-07	ELOVL fatty acid elongase 5
ALOX15B_1	-2,03	6,17	8,67E-04	1,75E-02	arachidonate 15-lipoxygenase, type B
IL36G_3	2,02	6,53	4,10E-08	7,87E-06	interleukin 36, gamma

KDM6B_1	-2,01	8,18	1,87E-23	4,00E-20	lysine (K)-specific demethylase 6B
DMKN_15	1,99	4,38	3,00E-11	1,61E-08	dermokine
MSMO1_2	-1,97	7,69	4,34E-07	5,73E-05	methylsterol monooxygenase 1
