# Supplementary material for: Psoriasis-Specific RNA Isoforms Identified by RNA-Seq Analysis of 173,446 Transcripts
Source: Front Med (Lausanne). 2016 Oct 7;3:46. doi: 10.3389/fmed.2016.00046 (PMC5053979; doi:10.3389/fmed.2016.00046)
Supplement: Table S3 — Differentially expressed transcripts in lesional skin compared to non-lesional skin (LP–NLP). [file Table_3.PDF]

Table 3S. Pair-wise analysis of transcript isoforms in psoriasis patients, LP-NLP comparison

Symbol	logFC	logCPM	LR	PValue	FDR	ENSEMBL ID	Gene name
S100A8_2	2,72	5,83	314,44	2,35E-70	6,68E-66	ENST00000368732	S100 calcium binding protein A8
KLK10_4	2,75	6,99	288,23	1,21E-64	1,71E-60	ENST00000358789	kallikrein-related peptidase 10
MPZL2_4	2,26	6,89	237,71	1,24E-53	1,17E-49	ENST00000278937	myelin protein zero-like 2
EHF_12	1,81	8,07	223,68	1,43E-50	1,01E-46	ENST00000257831	ets homologous factor
FABP5_3	3,19	6,62	196,26	1,37E-44	7,75E-41	ENST00000481695	fatty acid binding protein 5 (psoriasis-associated)
IL36RN_4	2,71	7,67	195,56	1,94E-44	9,19E-41	ENST00000393200	interleukin 36 receptor antagonist
PLA2G4D_2	3,24	5,87	186,26	2,08E-42	8,43E-39	ENST00000290472	phospholipase A2, group IVD (cytosolic)
LYZ_1	1,92	6,89	184,16	6,00E-42	2,13E-38	ENST00000261267	lysozyme
NAMPT_1	1,88	7,67	174,35	8,29E-40	2,61E-36	ENST00000222553	nicotinamide phosphoribosyltransferase
TGM3_1	2,02	7,89	173,42	1,33E-39	3,77E-36	ENST00000381458	transglutaminase 3
CNFN_2	2,78	7,11	167,01	3,33E-38	8,60E-35	ENST00000222032	cornifelin
RAB3B_1	-2,48	4,92	160,40	9,24E-37	2,18E-33	ENST00000371655	RAB3B, member RAS oncogene family
SLC5A1_2	1,95	6,89	160,15	1,05E-36	2,29E-33	ENST00000266088	solute carrier family 5 (sodium/glucose cotransporter), member 1
CTSC_4	2,11	6,23	157,14	4,78E-36	9,69E-33	ENST00000227266	cathepsin C
KLK13_1	2,63	3,87	156,57	6,36E-36	1,20E-32	ENST00000601975	kallikrein-related peptidase 13
SAMD9_1	2,02	6,54	152,73	4,39E-35	7,78E-32	ENST00000379958	sterile alpha motif domain containing 9-like
FAM43A_1	2,02	4,82	152,30	5,44E-35	9,09E-32	ENST00000329759	family with sequence similarity 43, member A
ADAMTSL3_1	-2,68	4,00	150,81	1,15E-34	1,82E-31	ENST00000286744	ADAMTS-like 3
ZC3H12A_2	2,44	5,19	148,45	3,79E-34	5,66E-31	ENST00000373087	zinc finger CCCH-type containing 12A
SGK1_16	1,73	6,68	146,48	1,02E-33	1,45E-30	ENST00000474427	serum/glucocorticoid regulated kinase 1
CDHR1_4	-1,82	7,83	146,06	1,26E-33	1,70E-30	ENST00000332904	cadherin-related family member 1
CDHR1_2	-2,52	8,46	145,21	1,94E-33	2,50E-30	ENST00000459673	cadherin-related family member 1
HIGD1A_1	1,88	6,44	144,49	2,78E-33	3,43E-30	ENST00000321331	HIG1 hypoxia inducible domain family, member 1A
FABP5_2	3,63	8,34	144,19	3,23E-33	3,82E-30	ENST00000297258	fatty acid binding protein 5 (psoriasis-associated)
ALOX12B_2	1,85	7,64	143,74	4,05E-33	4,60E-30	ENST00000319144	arachidonate 12-lipoxygenase, 12R type
